# Acute Lobar Pneumonia—Treatment Specially†Read before the Richmond Academy of Medicine and Surgery, January 26, 1897.

**Published:** 1897-03

**Authors:** Ramon D. Garcin

**Affiliations:** Richmond, Va., Instructor in Practice of Medicine, Medical College of Virginia; 2318 East Broad St.


### ACUTE LOBAR PNEUMONIA—TREATMENT SPECIALLY.†

BY RAMON D. GARCIN, M. D., RICHMOND, VA.,

Instructor in Practice of Medicine, Medical College of Virginia.

*Prognosis.*—Osler makes the statement, in his classical work, that "Pneumonia is one of the most fatal of acute diseases. Hospital statistics show that the mortality ranges from 20 to 40 per cent. Of 1,012 cases treated in the Montreal General Hospital, the mortality was 20 4 per cent. It appears to be somewhat more fatal in Southern climates. Statistics from the Charity Hospital, New Orleans, show the death-rate to be 28.01 per cent. It has been urged that the mortality in this disease has been steadily increasing, and attempts have been made to connect this increase with the expectant plan of treatment, but careful analysis of 1,000 cases by

*This case also bears out a statement made by Dr. Ashurt, in a recent article, in which he claims that the present methods of surgery in compound fractures, while greatly lessening mortality by the prevention of suppuration, does give in many instances delayed union. Here we have the bone in which suppuration occurred, giving firm union, and non-union in that in which it was prevented.

†Read before the Richmond Academy of Medicine and Surgery, January 26, 1897.

Townsend, in the Massachusetts General Hospital, indicates clearly that, when all circumstances are taken into consideration, the conclusion is not justified. He found that when all cases over fifty years of age were omitted, and those patients who were delicate, intemperate, or the subject of some complication, there was very little variation from decade to decade, and that, excluding these cases, the rate was little over 10 per cent. In answer to the assertion that the modified treatment is in part responsible for the increased mortality, he shows clearly that the rise in death-rate took place in the period prior to 1860, when the treatment was entirely or in great part heroic."

In a short interview with a prominent member of the professsion, he made the statement to me that in the discussion of the mortality of acute pneumonia, with Dr. Hunter McGuire, their experience with this disease was similar. They had never known a young adult whose previous history was good, to die with pneumonia. A careful study of my notes of cases shows that, while I have treated a good many cases in healthy young adults, my limited experience has been the same as theirs.

*Diagnosis.*—"No disease is more readily recognized in a large majority of cases. The external character, the sputa, and the physical signs combine to make one of the clearest of clinical pictures."—*Osler.* Dr. Osler says: "After a study in the post-mortem room, of my own and others' mistakes, I think that the ordinary lobar pneumonia of adults is rarely overlooked. Judging from my autopsy records, I should say that errors are particularly liable to occur in the intercurrent pneumonias, in those complicating chronic affections, and in the disease as met in children, the aged, and drunkards. Pleurisy, with effusion, is, I believe, not often mistaken, except in children."

*Treatment.*—Wood and Fitz: "In the treatment of pneumonia, it is essential to recognize that, though the disease may be a unit from the pathological point of view, therapeutically it comprises essentially diverse diseases. A pneumonia whose physical signs can not be made out in the beginning, but gradually creep up towards the chest wall, a pneumonia whose expectoration is, in the beginning, prune juice, whose crepitant rale is never typical, whose physical signs are obscure until complete consolidation gives percussion dullness, or a pneumonia occurring in the alcoholic, in the old, in the victim of renal disease, in the broken-down debauchee, is, in its management, essentially distinct from a pneumonia the result of exposure of a strong, healthy countryman to a Western blizzard or other cold." "When, in the first twenty-four hours of a pneumonia, there is violent constitutional reaction, with flushed face, rapid and noisy breathing, bloody sputa, intense headache and drowsiness, a hard, bounding, or a tense, corded pulse, venesection may markedly lessen all the symptoms, and, if combined with dry cupping over the chest, may, we believe, lessen the amount of engorgement of the lung and the final area of consolidation." Wet compresses, and even the application of the ice-bag very freely over the affected lung, are recommended by some of the leading text-books and authorities; and just at present, the ice treatment is quite a fad at the North. At the very beginning of an attack, where active congestion is just begun, ice might prove of service, as it is of value in any other form of active congestion; but, even then, it should be, it seems to me, used with the greatest caution, if at all, because of the shock, more or less, and the causes calling for its indication should have to be very carefully selected. Personally, I have had no experience with it, and shall certainly not use it, till I am fully convinced that the after-effects are not worse than the remedy.

"The question how far we should attempt to reduce temperature is important in the extreme, and is answered very differently in practice by different practitioners." Although a great many authorities advise against the use of any of the coal-tar class, my experience does not agree with their statement. When the temperature is 103°–4°, I use frequently, five grain doses of phenacetine, repeated at regular intervals, until the fever is reduced to 100° or so, and never, as yet, have I seen any but the best results follow its administration. Quinine, if used, has to be employed in large doses, and rather does harm than good, because of the resulting nervous excitation. I prefer the bisulphate or muriate.

"To prevent exhaustion by maintaining the forces of the patient, is the great object of the nursing in pneumonia. Absolute confinement in bed, from the beginning, regulating the temperature of the room 70° to 74°, feeding at regular intervals (two or three hours),—food should be simple, nutritious, and digestible; milk, animal broths, clam broth is very acceptable." Panopepton (Fairchild's) and peptonoids (Arlington Chem. Co.). "Alcohol in the beginning of sthenic pneumonia, is injurious. In the advanced stages of the disease, it being regarded therapeutically as allied to an infectious fever, it is of service, employed judiciously." "In the advanced stages of a severe pneumonia, tinct. digitalis, 5 to 15 minims at intervals of 4 to 6 hours, acts well as a heart stimulant. Its effects upon the pulse should be the guide for administering it."

Nitro-glycerin and amyl nitriate should be used with caution in sufficient doses to produce effects. They always lower the arterial pressure by depressing directly the muscle fibres in the blood-vessel's walls, and although their first action upon the heart is a stimulant, the slightest overdose converts such action into that of a powerful depressant. Carbonate of ammonium is very largely used, both as a stimulant and as an expectorant." Some authorities, among them one that I quote from very freely—Wood and Fitz—say, "There is no reason for believing that it has any effect on the consolidated lung, and that its power as a stimulant is certainly inferior." I must enter my humble protest against these assertions, for in several hundred cases of this disease I have treated in hospital and general practice, I can candidly say that I have found this drug of the utmost service, both as an expectorant and stimulant. My dosage is also at variance with theirs. They advise three grains or so; I employ five to ten grains, as indicated in the individual case.

Aromatic spirits of ammonia is also valuable as a cardiac stimulant when needed. The most important drug, in my hands, as a respiratory and circulating stimulant, is strychnine. "The special indication for the free use of this agent is cyanosis, with hurried breathing and other evidences of respiratory distress." "For relief of local pain, dry cups probably give quickest relief."

Blisters, the greatest single agent in treating this disease, have saved more lives than all the drugs in the materia medica. If used skillfully and when indicated, that is, in the beginning of the third stage, they will afford prompt relief from pain, aid the expectoration of the clogged lung, and very materially hasten convalescence.

In conclusion, allow me, from the limited time assigned me, to entirely disclaim originality for this paper. The great mass of it is quoted from authorities—Osler, and Woods and Fitz. I have given them due credit for all their quotations, which are taken literally and liberally from their works.

*2318 East Broad St.*

